# Toll-like receptors (TLRs) in the trained immunity era

**DOI:** 10.7554/eLife.106443

**Published:** 2025-09-02

**Authors:** Lena Alexopoulou, Magali Irla

**Affiliations:** 1 Aix Marseille University, CNRS UMR7280, INSERM U1104 Centre d'immunologie de Marseille-Luminy (CIML) Marseille France; Münster University Hospital Germany; Indian Institute of Science Education and Research (IISER) India

**Keywords:** Toll-like receptors, trained immunity, microbial infection, chronic inflammation, innate immunity

## Abstract

The long-term functional adaptation of innate immune cells following an initial stimulation, referred to as trained immunity or innate immune memory, enhances responsiveness and protection against secondary infections. Toll-like receptors (TLRs), an evolutionarily conserved family, recognize microbial-associated molecular patterns, initiating innate and adaptive immune responses. TLR signaling cascades induce the production of pro-inflammatory cytokines, antimicrobial peptides, and interferons, promoting pathogen clearance, while also driving epigenetic and metabolic reprogramming that enhances immune responses and protection to subsequent challenges. However, TLRs also recognize endogenous ligands contributing to chronic inflammation and autoimmune diseases. This review examines the role of TLRs and their various agonists in mediating trained immunity across diverse immune cell types, with an emphasis on their dual role in protecting against infections and chronic inflammation. It highlights recent clinical trials of TLR agonists as immunomodulatory agents and their therapeutic potential in infectious diseases and cancer. By providing an in-depth analysis of TLR-driven trained immunity, this review highlights the extensive influence of TLRs on immune cell populations and their implications for the development of novel, broad-spectrum immunotherapies.

## The concept of trained immunity/innate immune memory

The term ‘trained immunity’ or innate immune memory, introduced in 2011, is a phenomenon wherein cells of the innate immune system, such as monocytes, macrophages, or natural killer (NK) cells, undergo functional reprogramming after exposure to primary infections or vaccines, resulting in enhanced responses upon subsequent encounters with pathogens (Netea et al., 2020; Netea et al., 2011). Unlike the adaptive immune system, which relies on antigen-specific recognition by T or B cells, trained immunity refers to the nonspecific, enhanced innate immune responses. Trained immunity is mediated by transcriptional, epigenetic, and metabolic changes that allow the innate immune cells to respond more robustly to subsequent infections, even those caused by unrelated pathogens. Therefore, this concept challenges the conventional view that immune memory is exclusive to the adaptive immune system (Netea et al., 2011). It was first described in studies involving the Bacillus Calmette-Guérin (BCG) vaccine for tuberculosis, which enhanced protection against not only *Mycobacterium tuberculosis* but also other unrelated secondary infections, in particular with *Candida albicans* or *Schistosoma mansoni* (Garly et al., 2003). Similar observations were reported with other microbial agents, such as *C. albicans* and its wall component β-glucan, reinforcing the idea that trained immunity extends beyond pathogen-specific defense mechanisms (Kleinnijenhuis et al., 2012; Quintin et al., 2012). Importantly, this protection was also observed in athymic mice lacking T cells, demonstrating a T cell-independent mechanism that rather involves activated tissue macrophages (Bistoni et al., 1988; Van’twout et al., 1992). Viral infections also exhibit similar potential, with latent herpesvirus capable of enhancing resistance against the bacterial pathogens *Listeria monocytogenes* and *Yersinia pestis* in mice (Barton et al., 2007).

Tolerance, on the other hand, is the process by which overactivation of the immune system is prevented when exposed to persistent or high-dose stimuli. Acting as a protective mechanism, tolerance reduces the risk of chronic inflammation and autoimmune responses by instructing immune cells to ignore or suppress responses to nonthreatening stimuli. Thus, while trained immunity amplifies the immune response, tolerance serves as a regulatory mechanism to suppress or control it, with both processes playing crucial roles in maintaining appropriate and efficient immune responses.

## TLRs and signaling pathways

**Figure 1.**
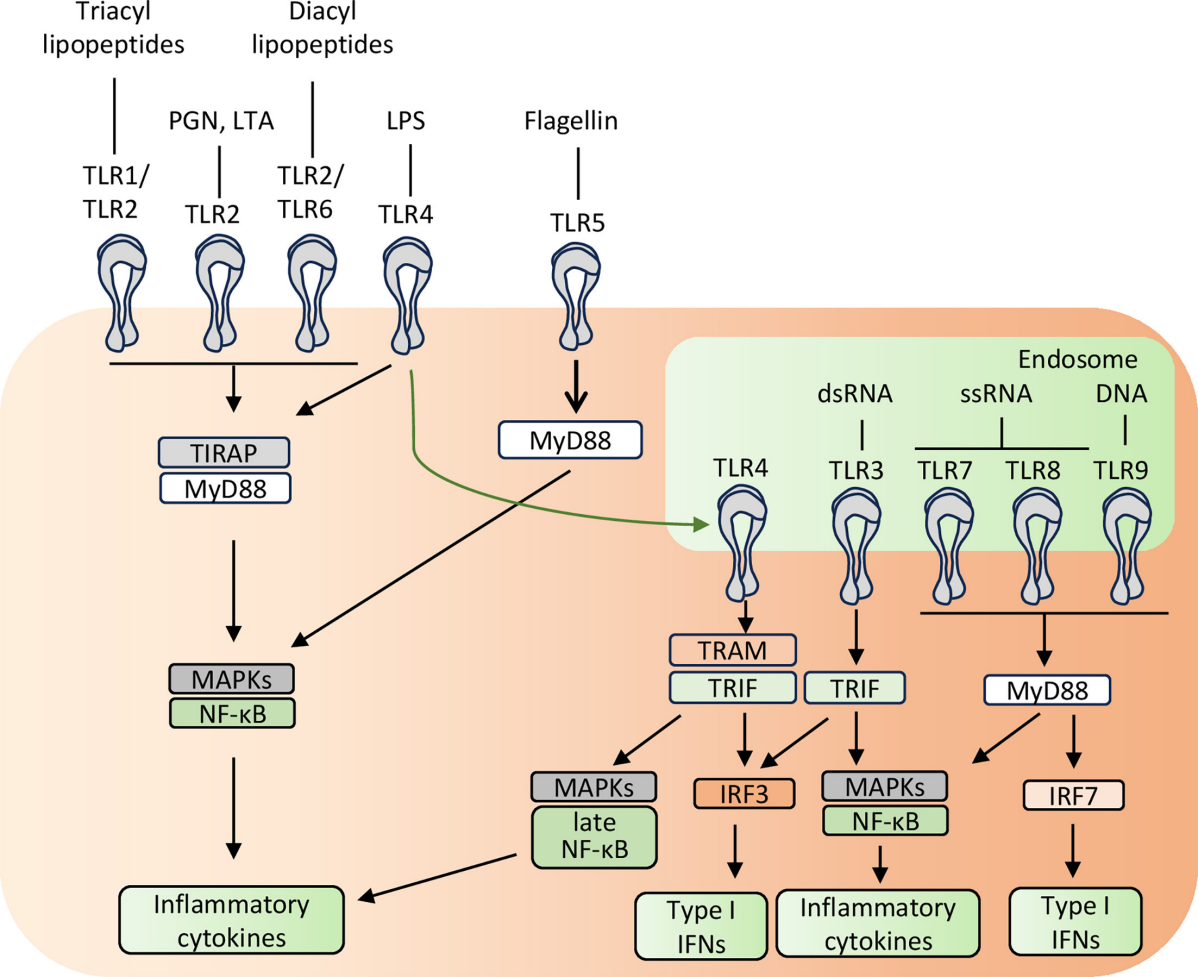
Toll-like receptor (TLR) microbial ligands and TLR-mediated immune responses. TLRs expressed on the cell surface recognize microbial membrane components, while TLRs expressed intracellularly in endosomal compartments recognize nucleic acids. Upon ligand binding, TLRs form homodimers or heterodimers and recruit adaptor molecules, such as MyD88 (myeloid differentiation primary-response protein 88), TIRAP (TIR-associated protein), TRAM (TRIF-related adaptor molecule), and/or TRIF (TIR domain-containing adaptor protein inducing IFN-β). Each TLR recruits a specific combination of adaptor molecules to activate downstream signaling cascades that lead to the production of inflammatory cytokines and type I IFNs.

Toll-like receptors (TLRs) are a family of evolutionary conserved innate immune receptors whose activation is pivotal for the induction of innate and adaptive immune responses (Kawai et al., 2024). TLRs can detect a vast range of microorganisms by recognizing highly conserved molecular patterns shared across various pathogen groups, commonly known as pathogen-associated molecular patterns (PAMPs). The TLR family consists of 10 members in humans (TLR1-TLR10) and 12 members in mice (TLR1-TLR9, TLR11-TLR13), with TLR1-TLR9 shared between both species, making them the most extensively studied. Each TLR recognizes distinct microbial components from viruses, bacteria, mycobacteria, fungi, or parasites and activates different signaling pathways (Figure 1). Based on their main cellular localization, TLRs can be classified as either cell surface or intracellular receptors. The cell surface TLRs mainly recognize microbial membrane components such as lipids, lipoproteins, and proteins. Indeed, TLR1, TLR2, and TLR6 recognize lipoproteins (Alexopoulou et al., 2002; Takeuchi et al., 1999; Takeuchi et al., 2001; Takeuchi et al., 2002) while TLR4 detects lipopolysaccharide (LPS) (Poltorak et al., 1998) and TLR5 senses flagellin (Feuillet et al., 2006; Hayashi et al., 2001). Intracellular TLRs are localized on endosomes and lysosomes and are specialized in nucleic acid (NA) recognition. TLR3 detects double-stranded RNA (dsRNA) (Alexopoulou et al., 2001), TLR7 and human TLR8 recognize single-stranded RNA (ssRNA) with distinct sequence preferences (Diebold et al., 2004; Heil et al., 2004; Lund et al., 2004), while TLR9 senses single-stranded DNA containing unmethylated CpG motifs (Hemmi et al., 2000). NA-sensing TLRs are particularly relevant for the detection of viruses. However, in certain cases, they may inappropriately respond to self-RNA released by damaged or stressed host cells, leading to the development of autoimmunity and autoinflammatory disorders, like systemic lupus erythematosus (SLE), Sjögren’s syndrome, or psoriasis (Alexopoulou, 2022; Hamerman and Barton, 2024; Sun et al., 2019).

TLRs are type I transmembrane proteins composed of a leucine-rich repeat ectodomain that mediates ligand recognition, a single transmembrane region, and a cytosolic Toll-IL-1 receptor (TIR) domain that activates downstream signaling pathways. Upon binding specific ligands, TLR ectodomains dimerize, leading to the dimerization of their cytosolic TIR domains that are recognized by two adaptor proteins: TIRAP (TIR-associated protein), also known as MAL, and TRAM (TRIF-related adaptor molecule) (Fitzgerald and Kagan, 2020; Kawai et al., 2024). Subsequently, TIRAP recruits the adaptor molecule MyD88 (myeloid differentiation primary-response protein 88), while TRAM recruits the adaptor molecule TRIF (TIR domain-containing adaptor protein inducing IFN-β), forming large oligomeric complexes that trigger cytosolic signaling. All TLRs, except TLR3, signal through MyD88, while TRIF is used by TLR3 and TLR4 (Fitzgerald and Kagan, 2020; Kawai et al., 2024). TRAM is recruited specifically to TLR4, but not TLR3, and serves as a link between TLR4 and TRIF. The combination of different adaptors engaged by each TLR determines the signaling pathways that will be activated. In general, MyD88 and TIRAP induce the activation of mitogen-activated protein kinase (MAPK)- and NF-κB-dependent pro-inflammatory responses, while TRAM and TRIF activate TBK1 and IκB kinase ε (IKKε) that are responsible for type I IFN production (Fitzgerald and Kagan, 2020; Kawai et al., 2024).

Determining whether MyD88 or TRIF is more critical for the immune response in a given cell type depends on TLR expression and downstream signaling, which together shape the strength and nature of the response. TLR4 is unique among TLRs in its ability to activate both the MyD88- and TRIF-dependent pathways, making it an ideal model for comparative analysis of these signaling cascades (Kawai et al., 2024). TLR4-MyD88 signaling is initiated at the plasma membrane, while TLR4-TRIF signaling occurs in endosomes, resulting in both spatial and temporal separation of these pathways. Studies using MyD88- and TRIF-deficient mice have shown that the relative contribution of these two pathways to TLR4-mediated responses is highly cell type-dependent. In macrophages, for instance, TLR4 activation by LPS revealed that TRIF signaling is crucial for upregulating costimulatory molecules and is strongly influenced by type I IFNs (Shen et al., 2008). In contrast, in DCs, neither MyD88 nor TRIF alone is required for this upregulation. Moreover, in both cell types, the MyD88 and TRIF pathways contribute collectively to the overall inflammatory response to LPS, but MyD88 plays a more important role than TRIF in T cell priming by LPS-matured DCs (Shen et al., 2008). In B cells, although both pathways contribute to lipid A moiety of LPS-induced B cell activation, MyD88 is more critical for CD86 upregulation and proliferation, whereas TRIF plays a unique role in class switch recombination and antibody production (Yanagibashi et al., 2015). Notably, studies in murine monocytes continuously exposed to varying LPS doses revealed that adaptation through the TLR4 pathway involves differential use of the TRIF and MyD88 signaling pathways. Specifically, prolonged low-dose LPS stimulation induces a TRIF-dependent inflammatory phenotype, whereas higher-dose stimulation drives a MyD88-dependent anti-inflammatory adaptation (Yuan et al., 2016). Thus, the contributions of MyD88 and TRIF pathways to TLR4-mediated immune responses are highly dependent on the cell type and agonist dosage, with each pathway playing distinct and complementary roles that shape the overall immune outcome.

The TLR signaling pathway, when excessively activated, is known to contribute to the onset and progression of autoimmune diseases, chronic inflammation, and even cancer (Wang et al., 2024). Therefore, to mitigate aberrant and excessive inflammatory responses, TLR signaling is regulated negatively by multiple intrinsic mechanisms, including disassembly of adaptor complexes, phosphorylation- and ubiquitin-mediated degradation of signal proteins, and transcriptional regulation, as summarized in the review by Kondo et al., 2012.

## Cell types capable of inducing trained immunity through TLR signaling

Trained immunity was initially described in monocytes and macrophages; however, it can be induced in a variety of cells, including neutrophils, innate lymphoid cells, NK cells, dendritic cells (DCs), endothelial cells, fibroblasts, epithelial cells, and hematopoietic precursors (Bekkering et al., 2021), all of which express various combinations and levels of TLRs. The primary role of TLRs is to protect the host from pathogens and thus can trigger the functional consequences of trained immunity that include pathogen clearance and cytokine production upon secondary challenge (Owen et al., 2020). Most cells express a limited combination of TLRs and often at low levels. However, innate antigen-presenting cells (APCs) such as monocytes, macrophages, DCs, and neutrophils tend to express many TLRs at relatively high levels. TLR expression levels can vary between different cell subtypes and may be significantly modulated upon their activation (Zarember and Godowski, 2002). The key to long-term memory in relatively short-lived immune cells, like neutrophils, relies on central trained immunity. Central trained immunity occurs in the bone marrow where hematopoietic stem cells (HSCs) undergo pro-inflammatory epigenetic reprogramming, which will be inherited by their daughter cells that will become trained effector innate immune cells (Ruffinatto et al., 2024). Indeed, HSCs and hematopoietic stem and progenitor cells (HSPCs) express functional TLR2, TLR4, TLR7, TLR8, and TLR9, which can regulate their proliferation, mobilization, and differentiation (Kim et al., 2005; Nagai et al., 2006; Sioud and Fløisand, 2007; Sioud et al., 2006; Yáñez et al., 2013). Contrary to central trained immunity, peripheral trained immunity occurs in differentiated innate immune cells, such as macrophages, DCs, and NK cells, in the bloodstream or peripheral tissues.

Although TLR agonists can trigger trained immunity and significantly enhance antimicrobial functions, the potential of different TLR agonists and their optimal dosing across various cell types to induce trained immunity has not been systematically studied. The study by Ifrim et al. examined how different doses of TLR agonists influence the development of trained immunity (Ifrim et al., 2014). This was addressed by stimulating primary blood-derived human monocytes with various TLR ligands (Pam_3_CSK_4_ for TLR2/1, polyinosinic-polycytidylic acid [poly I:C] for TLR3, LPS for TLR4, flagellin for TLR5, R848 for TLR7/8, and CpG for TLR9) at different doses. Their findings demonstrated that ligands inducing signaling through the surface-expressed TLR2, TLR4, and TLR5, as well as the intracellular TLR3 and TLR7/8, can induce trained immunity when used at appropriate low doses. In contrast, high doses of the same ligands led to tolerance (Ifrim et al., 2014). Additionally, TLR9 stimulation with low or high doses of CpG failed to induce trained immunity or tolerance. Thus, in human monocytes, both the nature and the concentration of the TLR agonists are critical determinants of trained immunity, with low doses of TLR ligands promoting the induction of trained immunity, whereas high doses favor the development of tolerance. Consistently, pretreatment of mice with low doses of LPS provided better protection against a lethal Sendai virus infection compared to high doses, an effect that was abolished in TLR4- or MyD88-deficient mice (Resiliac et al., 2023). Notably, most in vitro or in vivo studies investigating the ability of TLR agonists to induce trained immunity typically assessed only a single, often high, dose of the respective stimuli. This may be misleading, as low-dose TLR ligand stimulation is critical for inducing trained immunity (Ifrim et al., 2014). In this review, we will primarily focus on recent studies demonstrating TLR-induced trained immunity across various cell types in vitro, followed by in vivo studies.

### TLR1, TLR2, and TLR6 that detect lipoproteins and signal through MyD88 in trained immunity

TLR2 senses bacterial lipoproteins by forming heterodimers with either TLR1 or TLR6. The TLR2/TLR1 heterodimer recognizes triacyl lipoproteins, while the TLR2/TLR6 heterodimer is specialized for diacyl lipoproteins (Fitzgerald and Kagan, 2020; Kawai et al., 2024). TLR2/TLR1 and TLR2/TLR6 signal through the MyD88-dependent pathway, leading to the production of pro-inflammatory cytokines, although production of type I IFNs has also been reported (Fitzgerald and Kagan, 2020; Kawai et al., 2024).

Macrophage-activating lipopeptide-2 (MALP-2) is a mycoplasmal diacylated lipopeptide with immunomodulatory activity that induces TLR2/TLR6 dimerization and signaling (Seya and Matsumoto, 2002; Takeuchi et al., 2001). MALP-2 is able to activate a range of immune cells, including monocytes/macrophages, DCs, neutrophils, endothelial cells, and fibroblasts. It is a promising vaccine adjuvant with a wide range of immunological applications, such as wound and bone healing, vascular regeneration, and infection prevention (Liao et al., 2023). However, concerns have been raised about MALP-2’s specificity and safety in the human body, but given that TLR2 signaling exhibits promising immunomodulatory activity, several synthetic analogs of MALP-2, containing palmitoylated (Pam) peptides, have been generated and investigated.

Pretreatment of murine HSPCs with the TLR2/TLR1 agonist Pam3CSK4, followed by a second stimulation on day 7, resulted in APCs with enhanced expression of MHCII and costimulatory molecules, increased IL-12 production, and an improved capacity to prime T cells (Martínez et al., 2020). Moreover, APCs derived from HSPCs exposed to Pam3CSK4 showed enhanced Th1 and Th2 responses in CD4 T cell co-cultures upon subsequent stimulation with *C. albicans*. Thus, Pam3CSK4 detection by bone marrow HSPCs can drive trained immunity. However, in a different study, a single intravenous administration of Pam3CSK4 in mice, followed by in vitro differentiation of their HSPCs into macrophages in the presence of *C. albicans*, led to tolerance (Martínez et al., 2018).

The peptide Pam_2_Cys is a stable TLR2/TLR6 agonist with suitable characteristics for in vivo delivery (Tan et al., 2012). In mice, intranasal administration of Pam_2_Cys provides protection against viral infections such as influenza and rhinovirus, by reducing viral load, increasing survival, and promoting anti-inflammatory molecules, without compromising adaptive immune responses (Girkin et al., 2021; Mifsud et al., 2015; Tan et al., 2012). In the case of influenza infection, this protective effect is lost in TLR2-deficient mice (Tan et al., 2012). Additionally, intravenous administration of Pam_2_Cys in mice also safeguards against the malaria parasite *Plasmodium yoelii* by lowering the parasite burden in the liver (Ernest et al., 2018).

TLR2/6 signaling can enable the transmission of trained immunity across generations in murine offspring, inherited from parents that were challenged with zymosan or infected with *C. albicans* (Katzmarski et al., 2021). Zymosan, a polysaccharide originating from the yeast *Saccharomyces cerevisiae*, can be detected by TLR2/6, while TLR2 and TLR4 are involved in the host interaction with *C. albicans* and play a significant role in the development of host immune responses during candidiasis (Luisa Gil et al., 2016). Offspring of trained mice displayed cellular, transcriptional, and epigenetic changes in bone marrow myeloid cells and showed increased resistance to endotoxin challenge and systemic heterologous infections by *Escherichia coli* or *L. monocytogenes* (Katzmarski et al., 2021). Moreover, infection of male mice with the fungus *C. albicans* induced changes in sperm DNA methylation linked to immune gene loci (Katzmarski et al., 2021). These findings support the intergenerational inheritance of trained immunity in mammals, which enhances protection against infections, a phenomenon also observed in plants and the nematode worm *Caenorhabditis elegans* (Katz et al., 2009; Molinier et al., 2006). However, another study, using similar murine models of trained immunity induced by BCG vaccination, β-glucan, or *C. albicans* infection, did not provide protection against subsequent viral, bacterial, or fungal infections in the offspring of the trained parents (Kaufmann et al., 2022). Although the underlying cause of the discrepancy between the two studies remains undetermined, it could be related to differences in environmental variables, including the microbiome, mouse substrains, dietary factors, or the specific infection models employed (Katzmarski et al., 2022). Thus, whether trained immunity can be transmitted remains unclear and warrants further investigation.

### TLR4 that detects LPS and signals through MyD88 and TRIF in trained immunity

LPS, a compound found in Gram-negative bacteria, is a powerful immunomodulator commonly used to stimulate cells and model inflammatory responses. TLR4 recognizes LPS with the involvement of CD14, LPS-binding protein, and MD2 molecules, signals through both the MyD88- and TRIF-dependent pathway, driving the production of inflammatory cytokines and type I IFNs. It is widely recognized that pretreatment of mice with LPS provides a survival advantage against a variety of bacterial (Murphey et al., 2008; Varma et al., 2005), viral (Gu et al., 2023; Resiliac et al., 2023), and fungal infections (Rayhane et al., 2000), mainly by reducing the microbial load and inflammation. In humans, LPS instillation in lungs primes alveolar macrophages to subsequent ex vivo secondary stimulation to TLR2 or TLR4 agonists, as reflected by increased IL-1β and IL-6 expression (Hoogerwerf et al., 2010). Recent studies attempt to clarify in more detail how TLR4-mediated protective mechanism operates at the cellular, molecular, and genomic levels.

Microglia, the principal innate immune cells of the central nervous system, are capable of sensing environmental inflammatory mediators and neurotoxicants. Stimulation of a mouse microglia cell line with LPS, as a memory priming trigger, followed by manganese (a Parkinsonian-linked environmental neurotoxic stressor), as a secondary environmental trigger, resulted in enhanced immune responses, including increased production of IL-1α, IL-1β, IL-6, and TNF (Huang et al., 2023). LPS-induced microglia-retained immune memory was attributed to epigenetic reprogramming through histone H3K27 acetylation. Moreover, stimulation of murine microglia with TLR2/1 ligand Pam_3_CSK_4_, TLR4 ligand LPS, or TLR9 ligand CpG increased resistance against the encapsulated fungus *Cryptococcus neoformans* that can cause meningitis- or pneumonia-like symptoms (Redlich et al., 2013). Interestingly, in mice, peripheral administration of two low-doses of LPS induced immune stimulation and led to trained immune memory in the brain, predominately mediated by microglia and accompanied by elevated levels of TNF and IL-1β in the brain (Wendeln et al., 2018). However, when LPS was administered for 4 consecutive days, it led to immune tolerance. Notably, peripheral administration of a low dose of TNF also elicited trained immunity effects in the brain, whereas a high dose of LPS or IL-10 induced a tolerance effect (Wendeln et al., 2018). Acute immune training and tolerance in the brain lead to epigenetic reprogramming of microglia that persists for at least 6 months. In addition, using a mouse model of Alzheimer’s disease, it was shown that peripheral LPS-induced training promotes neuropathology, while LPS-induced tolerance attenuates it (Wendeln et al., 2018). Thus, while training in the periphery may be beneficial due to enhanced pathogen elimination, in the brain, it can lead to long-term modulation of microglial responses, potentially contributing to the severity of neurological diseases.

HSCs, corresponding to the progenitors of all blood and immune cells, can directly respond to infection and establish trained immunity through TLR signaling (Ruffinatto et al., 2024). In mice, a single LPS injection caused transient activation of HSCs and HSPCs, leading to persistent epigenetic changes in myeloid enhancers and innate immune system genes that lasted for at least 3 months (de Laval et al., 2020). Direct TLR4 signaling played a major role in establishing LPS-induced epigenetic memory in HSCs, as evidenced by the analysis of TLR4-deficient HSPCs. Additionally, transplantation of LPS- or poly I:C-trained HSCs into mice led to enhanced myeloid differentiation that protected against subsequent infection by the gram-negative bacterium *Pseudomonas aeruginosa*. Thus, a single LPS or poly I:C stimulation can induce innate immune memory in HSCs that can be transmitted to the progeny.

The side effects associated with the strong inflammatory response mediated by LPS limit its use as a clinical immunomodulator. Analogs of lipid A that show reduced pro-inflammatory activity but retain attractive immunomodulatory properties have therefore been developed (Bohannon et al., 2013). One such TLR4 agonist is monophosphoryl lipid A (MPLA), which is used clinically as a vaccine adjuvant (Mata-Haro et al., 2007). Several studies revealed that immunostimulation with MPLA induces trained immunity and provides protection against clinically relevant pathogens, such as Gram-negative *P. aeruginosa*, Gram-positive *Staphylococcus aureus*, fungal *C. albicans*, or influenza virus (Casilag et al., 2021; Fensterheim et al., 2018; Romero et al., 2011). In mice, MPLA-induced protection against *S. aureus* infection was lost in MyD88-deficient mice, but preserved in TRIF-deficient mice, with macrophages mediating the effect (Owen et al., 2022). Furthermore, CpG, which activates the MyD88 pathway, enhanced antimicrobial and metabolic activity in murine bone marrow-derived macrophages, whereas poly I:C, a TRIF-pathway agonist, did not. Notably, MPLA stimulation also induced similar protective macrophage reprogramming in human monocyte-derived macrophages (Owen et al., 2022). These findings suggest that MyD88-dependent signaling is critical for TLR-mediated trained immunity.

As an adjuvant, MPLA enhances the ability of macrophages and B cells to sensitize naïve T cells, and induces DC migration and maturation, supporting antigen-specific immune activation in vivo (De Becker et al., 2000). Therefore, MPLA serves as an adjuvant in vaccination studies against Zika virus (Shah et al., 2024), Rabies virus (Chen et al., 2019b), *Leishmania donovani* parasites (Mahapatra et al., 2024), and has been licensed as an adjuvant of the human papilloma virus vaccine (Garçon et al., 2011; Wang et al., 2020). The clinical outcome of MPLA and its potential to augment the efficiency of existing immunotherapeutic agents has led to the development of various novel synthetic TLR4 agonists that are explored for clinical translation (Hu et al., 2023; Kayesh et al., 2023; Rolfo et al., 2023).

### TLR5 that detects bacterial flagellin and signals through MyD88 in trained immunity

TLR5 detects bacterial flagellin proteins, the structural component of bacterial flagella and a virulent factor, and provides antibacterial host defense through MyD88 signaling (Feuillet et al., 2006). It is predominantly expressed on monocytes/macrophages and DCs, as well as on epithelial cells in various tissues. Flagellin is a potent immune activator, and as such, various studies have explored its adjuvant activity, either alone or as a fused protein, in combination with bacterial, viral, or parasitic antigens (Hajam et al., 2017). Flagellin-based adjuvants have shown in vivo protection against various types of infections, including *Y. pestis* (Mizel et al., 2009), *Streptococcus pneumoniae* (Lim et al., 2015; Muñoz et al., 2010), *Plasmodium falciparum* (Carapau et al., 2013), West Nile virus (McDonald et al., 2007), influenza virus (Hinkula et al., 2019; Kim et al., 2024), and SARS-CoV2 (Jiang et al., 2021). Interestingly, flagellin-based adjuvants have been shown to enhance protection against influenza in the elderly (Taylor et al., 2011) and pneumococcal infection in aged mice (Lim et al., 2015), as well as to improve longevity and health span in mice (Lim et al., 2024).

### TLR3 that detects dsRNA and signals through TRIF in trained immunity

TLR3 is an intracellular TLR that recognizes viral dsRNA and poly I:C, a synthetic analog of dsRNA (Alexopoulou et al., 2001; Wang et al., 2004). TLR3 signals through the TRIF-dependent pathway, leading to the activation of NF-κB, MAPKs, and IRF3 and the production of type I IFNs and inflammatory cytokines (Kawai et al., 2024). The affinity between TLR3 and dsRNA relies on the RNA length and the pH of the intracellular compartments where they are located (Kawai et al., 2024). Consequently, different lengths of poly I:C can induce distinct innate immune responses (Mian et al., 2013).

Pre-exposure of human fetal progenitor endothelial cells and differentiated human umbilical vein endothelial cells to poly I:C, followed by LPS stimulation, led to the training or tolerization of specific genes. Both cell types showed a strong overlap in their responses; however, fetal progenitor endothelial cells demonstrated a greater capacity to establish immune memory (Weiss et al., 2021). Moreover, pre-exposure of human lung fibroblast cell lines (MRC5 and HF19) to poly I:C, followed by TNF, as a second stimulus, led to increased IL-6 production (Yap et al., 2022). Thus, human lung endothelial cells and fibroblasts may be capable of retaining immune memory upon poly I:C stimulation, although further studies are needed to confirm this.

In neutropenic mice, pretreatment with poly I:C increased survival and enhanced bacterial clearance against *E. coli* meningoencephalitis. However, it did not confer a survival advantage in immunocompetent animals (Ribes et al., 2020). The poly I:C-mediated protection in neutropenic mice correlated with an increase of NK and microglia cells and higher levels of IFN-γ and RANTES that were sustained for 14 days after infection (Ribes et al., 2020). In aged mice, intranasal administration of poly I:C provided protection against infection with either a mouse-adapted respiratory syndrome coronavirus (MA15) or influenza A virus, both of which cause severe disease in untreated aged mice (Zhao et al., 2012). The increased survival of the poly I:C pretreated mice was characterized by reduced viral load and increased DC migration and IFN-β, IFN-γ, IL-1β, and TNF levels in the lungs. Moreover, intranasal pretreatment with IFN-β or IFN-γ, but not IL-1β or TNF, also conferred protection in aged mice, supporting the hypothesis that the protective effect of poly I:C pretreatment is mediated, at least in part, through the induction of IFN-β and IFN-γ (Zhao et al., 2012). In a study on turbot (*Scophthalmus maximus*), feeding turbot larvae with poly I:C and subsequently challenging them with *Edwardiella piscicida* bacteria, a fish pathogen that primarily infects its host via the gastrointestinal tract, significantly enhanced survival rates by reducing bacterial load through a robust inflammatory response (He et al., 2021). Notably, the protective effect induced by poly I:C persisted for up to 7 weeks, indicating its potential as a long-lasting immunostimulant for the prevention of bacterial disease in aquaculture systems.

Poly I:C is an attractive adjuvant candidate for vaccines against intracellular pathogens or tumors, due to its ability to enhance T cell and antibody responses (Navabi et al., 2009; Ngoi et al., 2008). However, its instability and toxicity led to the generation of various derivatives that are currently under investigation in experimental, preclinical, and clinical studies (Komal et al., 2021; Matsumoto et al., 2020).

### TLR7 and TLR8 that detect ssRNA and signal through MyD88 in trained immunity

TLR7 and human TLR8 detect ssRNA of viral and bacterial origin, but with distinct ligand specificities (Tanji et al., 2015; Zhang et al., 2018). These receptors exhibit different expression patterns, with human TLR7 mainly expressed on plasmacytoid DCs (pDCs) and B cells, whereas TLR8 is predominately expressed in myeloid cells (Kawai et al., 2024). In contrast, murine TLR8 is unable to detect ssRNA due to the absence of five amino acids in its extracellular domain that are critical for RNA recognition (Liu et al., 2010). Consequently, our current understanding of TLR7 biology is considerably more advanced than that of TLR8. Importantly, both TLR7 and TLR8 can also recognize self-RNA, and their dysregulated signaling has been implicated in autoimmune diseases (Hamerman and Barton, 2024).

Imiquimod (R837), an imidazoquinoline amine analog to guanosine, is a TLR7 agonist with potent antiviral and immunomodulatory activity. Clinically, it is used for the topical treatment of abnormal skin growths, including genital warts and superficial basal cell carcinoma. Pretreatment of human bronchial epithelial cells with imiquimod triggered viral resistance to SARS-CoV-2 virus by decreasing angiotensin-converting enzyme 2, the receptor of SARS-CoV-2, and increasing IFN-β expression (Nieto-Fontarigo et al., 2021). In the case of respiratory syncytial virus (RSV) infection, imiquimod reduced cytokine production in RSV-infected epithelial cells, while in a murine model of RSV infection, it provided protection by reducing RSV lung titers, airway inflammation, and weight loss (Salinas et al., 2020). Similarly, intranasal or epicutaneous pre-administration of imiquimod protected mice against influenza A infection (To et al., 2019). Remarkably, imiquimod and BBIQ (1-benzyl-2-butyl-^1^H-imidazo[4,5-*c*] quinolin-4-amine), another pure TLR7 agonist, can act as influenza vaccines adjuvants (Bricha et al., 2023; Kaushik et al., 2020; Miller et al., 2020). Moreover, hepatocyte-targeted delivery of imiquimod by lipid-based nanoparticles in an experimental hepatitis B virus (HBV) mouse model led to increased IFN-α levels in the liver, which subsequently reduced serum levels of HBV surface antigen. This finding highlights its potential for treating chronic hepatitis B (Al Fayez et al., 2022), which is consistent with previous studies (Hu et al., 2020; Wang et al., 2014).

Application of the TLR7 agonist imiquimod on mouse skin can induce inflammation (Rabeony et al., 2015), during which epithelial stem cells acquired an inflammatory memory phenotype, leading to faster skin recovery when wounding was used as a second skin injury (Naik et al., 2017). This enhanced wound healing effects after skin inflammation was also observed with other treatments that induce atopic dermatitis, hyperplasia, epidermal abrasion wounds, or fungal infection with *C. albicans* (Naik et al., 2017). In the case of imiquimod-induced skin inflammation, the trained memory of the epithelial stem cells was linked to modifications in chromatin accessibility of genes associated with hyperproliferation and inflammation (Naik et al., 2017). Moreover, pathway analysis revealed that AIM2 and its downstream effectors, caspase 1 and IL-1β, were central regulators of the heightened wound-repair response seen in inflammation-experienced skin, and blockage of these molecules reversed the protective effect. However, the study did not assess the role of the MyD88 pathway, so the contribution of TLR7 signaling to this phenomenon remains unknown. Thus, once skin is sensitized to inflammation, it can react faster to a second assault.

Opioid use disorders and fatal overdoses, largely driven by fentanyl and its analogs, are prompting the exploration of vaccines as a long-lasting protective strategy against opioid exposure. As such, a synthetic TLR7/8 agonist INI-4001, but not the TLR4 agonist INI-2002, into a fentanyl-based conjugate vaccine significantly enhanced the production of high-affinity antibodies and reduced fentanyl distribution to the brain in mice, supporting its potential for further clinical development (Miller et al., 2023).

### TLR9 that detects single-stranded DNA and signals through MyD88 in trained immunity

The intracellular TLR9 recognizes DNA containing unmethylated CpG motifs derived from bacteria and viruses, as well as synthetic CpG oligonucleotides. It signals through the MyD88-dependent pathway and triggers NF-κΒ- and IFN-mediated pro-inflammatory responses. In humans, TLR9 is predominately expressed on pDCs and B cells and to a lesser extent on some subsets of macrophages and monocytes, which are crucial for inducing trained immunity. Moreover, TLR9 activation is more associated with immediate inflammation rather than the controlled and sustained immune enhancement needed for trained immunity. Consequently, TLR9 has been explored as a candidate for trained immunity induction. In particular, TLR9 stimulation of blood-derived human monocytes with low or high doses of CpG failed to induce trained immunity or tolerance, unlike other agonists such as TLR2, TLR3, TLR4, or TLR7 agonists which triggered trained immunity at low doses (Ifrim et al., 2014).

Nevertheless, studies conducted in mice have shown that CpG administration provides protection against a broad spectrum of viral, bacterial, and certain parasitic pathogens (reviewed in Krieg, 2007). In addition, a recent study revealed that in mice, CpG-induced protection against *S. aureus* infection relies on macrophage reprogramming, with the augmentation of macrophage antimicrobial functions occurring in parallel with their metabolic reprogramming (Owen et al., 2022). This protection was abrogated in MyD88-deficient, but not in TRIF-deficient, mice, suggesting that the MyD88-dependent signaling pathway is critical for CpG-mediated trained immunity.

## TLR-mediated trained immunity upon BCG vaccination

BCG vaccine, a live-attenuated mycobacteria-based vaccine, is a well-studied inducer of trained immunity (Chen et al., 2023). BCG vaccination protects against tuberculosis and has shown beneficial effects against respiratory viral infections, cancer (mainly bladder cancer and melanoma), autoimmune diseases (such as type 1 diabetes), and other diseases (Chen et al., 2023; Covián et al., 2019).

In humans, BCG-mediated trained immunity induces functional and epigenetic reprogramming of mature monocytes/macrophages (Arts et al., 2018; Li et al., 2023), HSPCs in the bone marrow (Cirovic et al., 2020), NK cells, and neutrophils (Moorlag et al., 2020). The immune response induced by BCG vaccination begins by the recognition of the bacillus by macrophages, DCs, and neutrophils at the inoculation site, through PAMPs, with TLRs playing an essential role. Indeed, MyD88-deficient mice show increased susceptibility to mycobacterial infection (Fremond et al., 2004; Scanga et al., 2004). The role of individual TLRs in the BCG response was studied in vitro by infecting murine bone marrow-derived macrophages and DCs from TLR2-, TLR3-, TLR4-, TLR7-, TLR9-, double TLR2/TLR4- and triple TLR2/TLR4/TLR9-deficient mice with *Mycobacterium bovis*. Analysis of costimulatory molecules and cytokines revealed that among the tested TLRs, TLR2 signaling played the most essential role in fighting against infection (Von Meyenn et al., 2006). This is in accordance with previous studies that have shown that TLR2 recognizes a lipoprotein and lipomannan from mycobacteria (Brightbill et al., 1999; Quesniaux et al., 2004; Thoma-Uszynski et al., 2001). Moreover, IL-12 production is also diminished in TLR2/TLR4/TLR9-deficient DCs, suggesting that TLR4 and TLR9 may also contribute to immune responses against *M. bovis* infection (Von Meyenn et al., 2006). Furthermore, studies with THP1 macrophages showed that *M. tuberculosis* can be sensed by TLR2 and TLR4 through the detection of the immunogenic Rv2627c and Rv2628 proteins, which become activated during latency and help the bacterium remain dormant (Bhatt et al., 2022).

In vitro studies with human monocytes revealed that heat-killed *M. tuberculosis* (HKMtb) can induce trained immunity by increasing the inflammatory response upon secondary stimulation with TLR4 (LPS) or TLR7/8 (R848), but not TLR2/TLR1 (Pam_3_CSK_4_) or TLR9 (CpG) ligands (Minute et al., 2024). This HKMtb-induced trained immunity relied on epigenetic reprogramming of hypoxia-induced factor 1α and Syk kinase. Additionally, in vivo studies showed that systemic or intranasal HKMtb administration enhances the immune response in mice to a heterologous LPS challenge, promoting a stronger pro-inflammatory profile (Minute et al., 2024).

Trained immunity can be induced not only by BCG vaccination, but also by various other vaccines, including those for influenza (Debisarun et al., 2021; Wimmers et al., 2021), measles (Röring et al., 2024), and COVID-19 (Baydemir et al., 2024; Murphy et al., 2023). A 6-month follow-up study showed 68% relative reduction in the risk of developing COVID-19 in volunteers aged 50 years or older who were vaccinated with BCG, compared to those in the placebo group (Tsilika et al., 2022). However, the role of TLR signaling in the induction of trained immunity mediated by these various vaccines remains poorly understood.

## TLR-mediated trained immunity by endogenous stimuli

Not only exogenous microbial components, but also endogenous stimuli such as endogenous retroviruses (ERVs), autoimmunity, sterile inflammation, certain diets and lipids, as well as damage-associated molecules can induce trained immunity through TLR signaling. ERVs comprising a substantial fraction of the mammalian genome, 8% in humans and 10% in mice, are typically silenced by epigenetic mechanisms but can be reactivated by several stimuli, including viral infections (Kassiotis, 2023). In mice, TLR7-driven antibody production serves as a key immune immunosurveillance mechanism against ERVs, while TLR3 and TLR9 provide complementary roles in preventing ERV-associated malignancies (Rauch et al., 2024; Young et al., 2012; Yu et al., 2012). In chicken embryonic fibroblasts, an antisense long noncoding RNA from the endogenous avian leukosis virus in chromosome 1 can induce antiviral defense responses by activating the TLR3 signaling pathway (Chen et al., 2019a). Moreover, a recent study analyzing large-scale transcriptomic and epigenetic data from human trained or tolerant macrophages revealed that specific human ERVs are actively transcribed, associated with gene regulation, and linked to inflammatory and immune response pathways, including the TLR pathway, highlighting their potential role in immune training (Du et al., 2024).

SLE is a chronic autoimmune disease that affects multiple organs and is characterized by the production of autoantibodies against nuclear antigens, with TLR7 signaling playing a pivotal role in its development (Kalliolias et al., 2024). Human monocytes stimulated with SLE-typical nuclear antigens, such as apoptotic microparticles, neutrophil extracellular traps, or plasma from SLE patients, and subsequently restimulated with TLR agonists (LPS or Pam_3_CSK_4_) exhibited increased pro-inflammatory cytokine production, along with transcriptomic and epigenetic changes, including an increase in histone 3 lysine 4 trimethylation (Yanginlar et al., 2024). Furthermore, circulating monocytes from SLE patients produced increased levels of pro-inflammatory cytokines after stimulation with TLR ligands. Thus, trained immunity may contribute to chronic inflammation in SLE, driven by persistent exposure to nuclear antigens. This process is also relevant in other autoimmune diseases, such as rheumatoid arthritis and multiple sclerosis (Funes et al., 2022; Mora et al., 2023).

Duchenne muscular dystrophy (DMD) is a severe, X-linked recessive disorder that primarily affects males. It is caused by mutations in the dystrophin gene, which prevent the production of dystrophin, a protein essential for muscle strength and stability, leading to progressive muscle weakness and premature death (Bez Batti Angulski et al., 2023). Inflammation is a hallmark of DMD, with TLR4 signaling playing a central role, since its genetic or pharmacological inhibition has been shown to improve muscle function and reduce pathology in mice (Bez Batti Angulski et al., 2023; Giordano et al., 2015). Notably, a recent study found that bone marrow-derived macrophages from a DMD murine model exhibited features of trained immunity, characterized by heightened inflammatory responses driven by metabolic and epigenetic changes. These alterations, induced by dystrophic muscle extract in a TLR4-dependent manner, were transmissible via bone marrow transplantation and persisted long-term (Bhattarai et al., 2022), suggesting that TLR4-dependent innate immune memory established in the bone marrow contributes to the dysregulated chronic inflammation in DMD.

Metabolic stimuli released by certain diets can also lead to trained immunity. It was reported that obesity induced by high-fat diet in early life causes long-lasting innate immune reprogramming, even after metabolic normalization. Stearic acid was the most abundant fatty acid bound to plasma phospholipids in mice fed a high-fat diet. Acting through TLR4, it was sufficient to trigger chromatic remodeling and enhance the accessibility of activator protein-1 (AP-1) (Hata et al., 2023). This shift promoted glycolysis in myeloid cells, driving pro-inflammatory cytokine expression, worsening retinal angiogenesis, and contributing to neuronal degeneration and vision loss (Hata et al., 2023). Additionally, a ketogenic diet enriched in saturated fatty acids induced trained immunity, leading to a heightened inflammatory response and increased mortality upon systemic LPS challenge in mice (Seufert et al., 2022). Palmitic acid was identified as a key mediator driving ceramide-dependent hyperinflammation, highlighting the impact of dietary saturated fatty acids on innate immune memory (Seufert et al., 2022). An in vitro study using human monocytes demonstrated that a brief exposure to low concentrations of oxidized low-density lipoprotein (oxLDL), followed by stimulation with TLR2 (Pam_3_Cys) or TLR4 (LPS) agonists, resulted in increased production of proatherogenic proteins and foam cell formation. This response was accompanied by elevated trimethylation of histone 3 lysine 4 (H3K4me3) histone mark, suggesting that oxLDL induces trained immunity in human monocytes, which may affect the development of atherosclerosis (Bekkering et al., 2014).

## Epigenetic and metabolic reprogramming by TLRs

**Figure 2.**
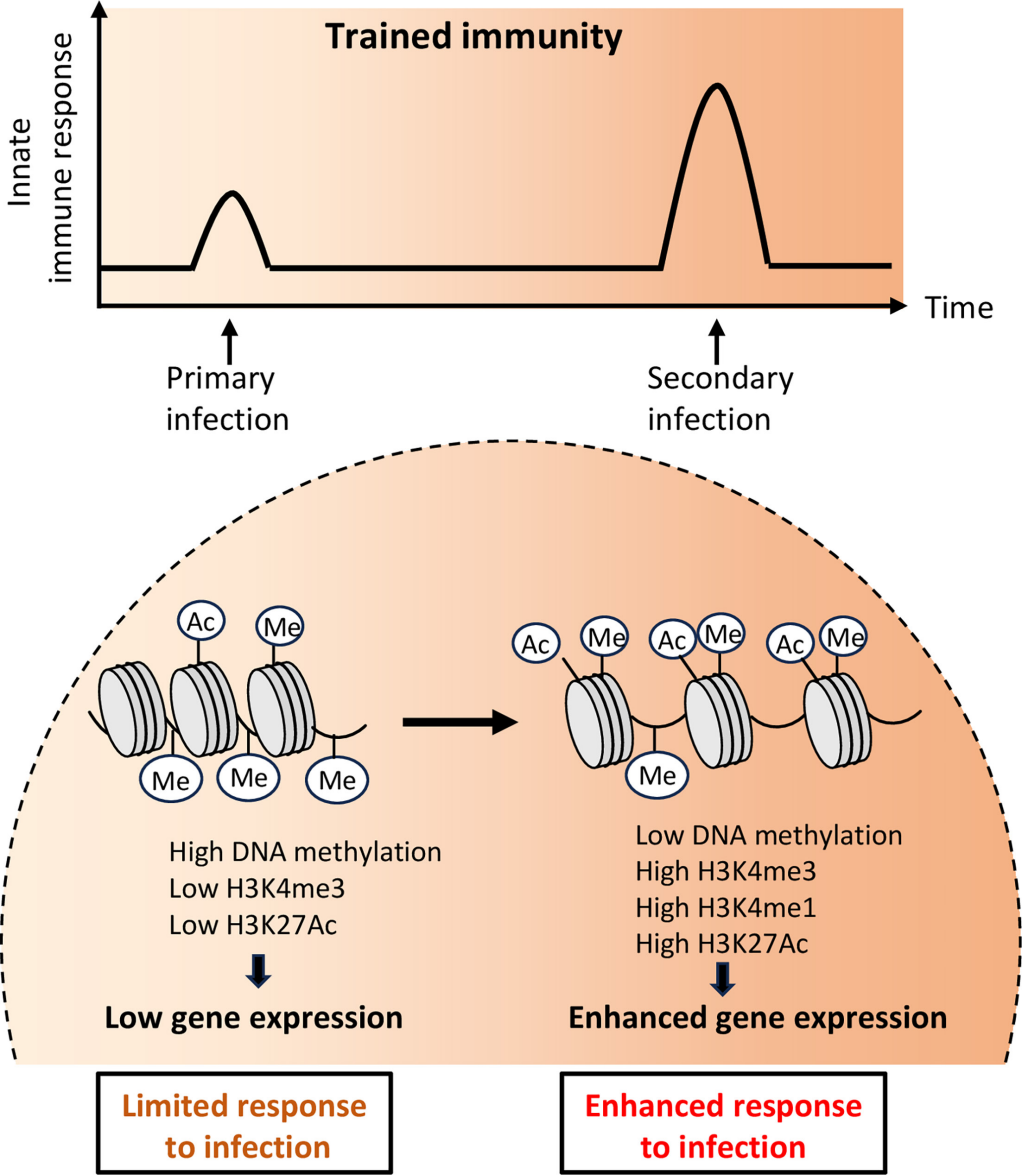
Trained immunity, mediated by epigenetic reprogramming, confers enhanced protection against secondary infection. A primary infection induces epigenetic reprogramming of activated genes in innate immune cells, enabling an amplified response to subsequent infections over time. This is characterized by a reduced DNA methylation status and the deposition of histone chromatin marks. These epigenetic changes persist partially after the primary stimulus ceases, facilitating rapid transcription of pro-inflammatory genes during re-infection. H3K4me, histone 3 lysine 4 methylation; H3K4me1, histone 3 lysine 4 mono-methylation; H3K27ac, histone 3 lysine 27 acetylation.

One of the key mechanisms through which TLRs contribute to trained immunity is by initiating epigenetic reprogramming (Figure 2). The first evidence of memory features linked to TLR-induced chromatin modifications was observed in mouse macrophages stimulated by LPS through TLR4 (Foster et al., 2007). In this study of LPS tolerance, gene-specific chromatin modifications were observed, which were associated with the silencing of inflammatory genes and increased transcription of genes encoding antimicrobial molecules. Upon TLR activation, various transcription factors (e.g. NF-κB, AP-1) are mobilized, which induce changes in chromatin structure at the promoter and enhancer regions of genes involved in inflammation and immune activation. For example, the TLR2 agonist MALP-2 and CpG agonist for TLR9 induced histone acetylation at a specific subset of poised enhancers in mouse macrophages, which enabled a faster and stronger response upon restimulation (Ostuni et al., 2013). TLR signaling triggers histone modifications, including increased trimethylation at histone H3 lysine 4 (H3K4me3) and acetylation at histone H3 lysine 27 (H3K27ac), enhancing the accessibility of these loci for transcription. These modifications act as ‘molecular bookmarks’ in the genome, enabling a more rapid transcriptional response upon restimulation. Additionally, TLR-induced DNA methylation changes in immune-related genes further contribute to the stable, heritable nature of this trained state, solidifying the epigenetic basis of trained immunity.

Recent studies highlight the crucial role of metabolic processes, especially aerobic glycolysis and tricarboxylic acid (TCA) cycle metabolites, in sustaining long-term epigenetic changes during trained immunity (Riksen and Netea, 2021). Upon the first stimulation of the immune system by pathogen- or danger-associated components, these metabolites act as a source of nutrients, as well as cofactors for epigenetic enzymes, driving chromatin remodeling and enhancing immune responses. This metabolic-epigenetic interaction ensures an enhanced response upon subsequent heterologous stimulation.

Glycolysis is the first step in the conversion of glucose into pyruvate, which can either be used for oxidative phosphorylation to generate ATP by entering the mitochondria for the TCA cycle (also known as the Krebs cycle) or be converted into lactate. While lactate production is common in low-oxygen conditions, it can also occur when energy demands exceed the capacity of oxidative metabolism, providing a rapid, but less energy-efficient source than oxidative phosphorylation. In trained immunity, glycolysis remains highly active even with sufficient oxygen, a phenomenon called aerobic glycolysis (also known as the Warburg effect), which serves as the metabolic foundation for trained immunity by supplying the necessary energy and substances for enhanced immune cell activation (Domínguez-Andrés et al., 2023). Initial studies investigating the metabolic reprogramming of trained immunity-mediated protection against infections using the fungal cell wall component β-glucan as stimuli in human monocytes revealed that increased aerobic glycolysis, glutaminolysis, and cholesterol synthesis are all important metabolic processes, whereas blockage of these pathways impaired the induction of trained immunity (Arts et al., 2016; Cheng et al., 2014). Additionally, subsequent studies with β-glucan, BCG, or oxLDL as primary stimuli revealed that various TCA intermediates exert distinct effects on innate immune cell activity, a topic that has been extensively discussed in recent reviews (Domínguez-Andrés et al., 2023; Vuscan et al., 2024).

Despite the critical role of TLR signaling in initiating trained immunity, the molecular mechanisms underlying TLR-mediated metabolic reprogramming remain incompletely characterized and have been investigated in only a limited number of studies. To evaluate if an increase in glycolysis and a decrease in oxidative phosphorylation is a general characteristic of monocytes that have encountered a pathogen, Lachmandas et al. stimulated human monocytes with LPS or Pam_3_CSK_4_. Although both TLR agonists increased glycolysis, only TLR4 activation by LPS led to a decrease in oxidative phosphorylation, while TLR2/6 activation by Pam_3_CSK_4_ led to increased oxidative phosphorylation and mitochondrial enzyme activity, suggesting that different TLR signaling pathways can induce distinct metabolic programs (Lachmandas et al., 2016).

However, in mice, treatment with MPLA led to enhanced resistance to subsequent infection with *S. aureus* or *C. albicans*, whereas tissue macrophages were required for the beneficial effects of MPLA (Fensterheim et al., 2018). Furthermore, treatment of macrophages with MPLA increased anaerobic glycolysis, as well as mitochondrial oxidative phosphorylation and mitochondrial biogenesis (Fensterheim et al., 2018). The discrepancy between these two studies concerning the nature of metabolic reprogramming induced by TLR4 signaling may stem from variations in agonists used and their concentration, as well as differences in cell type and species-specific responses.

Notably, a recent study that examined the contribution of long-term HSCs to trained immunity in the setting of autoimmunity revealed that the epigenetic and metabolic properties of trained immunity could be decoupled (Mills et al., 2024). As a chronic autoimmune model, they used the pristane-induced mouse model of SLE, which is TLR7-dependent (Savarese et al., 2008), and found that bone marrow macrophages from these mice exhibited hallmark features of trained immunity, such as enhanced bacterial killing and inflammatory cytokine production, linked to increased glycolysis. However, while HSCs serve as a long-term reservoir for trained immunity-associated macrophages, these cells displayed reduced glycolytic activity and chromatin accessibility at metabolic genes, suggesting a decoupling of functional and metabolic properties in trained immunity driven by autoimmune inflammation (Mills et al., 2024).

## Clinical trials on TLRs as immunomodulators

TLR agonists are emerging as promising tools in vaccine development and cancer immunotherapy (Anwar et al., 2019; Jeon et al., 2024; Mukherjee et al., 2023). TLR agonists are used to boost vaccine efficacy by promoting stronger immune responses to antigens. However, the challenge lies in balancing immune activation with minimizing adverse effects, as excessive stimulation can lead to inflammation or autoimmunity. Despite this, early-phase trials indicate that TLR agonists are generally well tolerated. Vesatolimod (also known as GS-9620), a potent TLR7 agonist small molecule, has been studied in clinical trials for its potential use in chronic viral infections such as HIV and HBV. In the case of HBV, which is a main cause of end-stage liver disease, including cirrhosis and hepatocellular carcinoma, Vesatolimod is assessed as part of combination therapies aimed at stimulating antiviral immune responses and reducing the viral load or clearing infection. For instance, the clinical trial NCT02579382 has focused on evaluating Vesatolimod in combination with tenofovir disoproxil fumarate for patients with HBV infection (Agarwal et al., 2018). This phase 2 trial was designed to investigate the efficacy and safety of Vesatolimod in stimulating immune responses, particularly its ability to reduce viral load and induce seroconversion (loss of HBeAg and HBsAg markers). Although Vesatolimod was safe and well tolerated and stimulated the expression of interferon-stimulated genes, it did not result in a significant HBsAg decline. Another phase 2 clinical trial (NCT02166047) showed that orally delivered Vesatolimod was safe and well tolerated in chronic HBV patients who were already virally suppressed (Boni et al., 2018; Janssen et al., 2018). This study showed no differences in serum levels of hepatitis B surface antigen but reported enhanced T-cell and NK-cell responses, suggesting that Vesatolimod may ameliorate the immune response against HBV and therefore contribute to the management of chronic viral infections.

In the case of HIV, the efficacy of Vesatolimod is assessed for its ability to activate the immune system to target and eliminate latent HIV reservoirs, with the aim of achieving a functional cure or ‘HIV remission’. For instance, the Phase 2a clinical trial, NCT05281510, is designed to evaluate the safety and tolerability of a combination of two anti-HIV envelope neutralizing antibodies, VRC07-523LS and CAP256V2LS, alongside the TLR7 agonist Vesatolimod, in women infected with HIV clade C who were treated early with antiretroviral therapy. This trial explores whether this combination can enhance immune responses and help in controlling the virus more effectively. It therefore investigates the extent to which this combination can help in maintaining viral suppression and reducing the HIV reservoir in early-treated patients.

The Phase 1/2a clinical trial, NCT06071767, is also designed to evaluate the safety, tolerability, and efficacy of a therapeutic vaccination regimen for HIV-1. This study investigates a conserved-mosaic T-cell vaccine based on chimpanzee adenovirus and the modified vaccinia Ankara platform, administered alongside Vesatolimod and two neutralizing antibodies.

Additional clinical studies are needed to determine the long-term efficacy and safety of Vesatolimod. These studies are expected to demonstrate whether they can induce immune responses without causing excessive inflammation, which is crucial to avoid autoimmune-like side effects. Other TLR agonists, in particular TLR9 agonists MGN1703 and CpG-7909, have been tested in the context of HIV infection. The clinical trial NCT00562939 investigated the safety and immunogenicity of adding a CPG 7909, as an adjuvant to pneumococcal vaccines in HIV-infected adults (Offersen et al., 2012; Søgaard et al., 2010). This study showed that the use of CPG 7909 significantly improved the immune response to the vaccine, although it also caused flu-like symptoms in a majority of participants receiving the adjuvant. These trials provided proof of concept that TLR9 agonists could be used to boost vaccine efficacy in HIV immunocompromised individuals. Moreover, several clinical trials have also shown that TLR agonists, such as TLR7 and TLR9, can promote robust antitumor responses (Le Naour and Kroemer, 2023; Rolfo et al., 2023). These agonists can activate DCs and boost T-cell activation, leading to more effective immune responses against cancer cells. For instance, TLR9 agonists such as CpG oligonucleotides used in combination with chemotherapy or immune checkpoint inhibitors have resulted in promising results characterized by a prolonged patient survival with melanoma or lymphoma. Phase 1 and Phase 2 clinical trials based on the use of TLR agonists for therapeutic intervention of human cancers, including melanoma, brain tumors, breast cancer, and prostate cancer, have been recently reviewed (Le Naour and Kroemer, 2023; Mukherjee et al., 2023).

In summary, clinical trials indicate that TLR agonists hold potential in both infectious diseases and oncology, especially when combined with other immunotherapies. However, key aspects of TLR agonist-mediated protection have yet to be clarified, such as the duration of protection and possibility of prolonging it with repeated treatments. Given that TLR stimulation triggers inflammatory pathways, it is also crucial to carry out dosing studies to avoid any potential risk of inflammation or autoimmune diseases. Identifying the most practical route of administration, likely via mucosal-based immunization, such as oral or intranasal (Kwong et al., 2023; Yusuf and Kett, 2017), and defining the appropriate dosage for various patient groups, including the elderly and individuals with comorbidities, are also crucial. Therefore, further investigations are needed to fully understand the underlying mechanisms of TLR agonists and optimize their clinical use.

## Negative regulators of trained immunity

Trained immunity enhances broad protection against infections, but its dysregulation can drive inflammatory diseases. Negative regulation of trained immunity is thus essential to maintain balance and prevent pathology. Two complementary studies revealed that the anti-inflammatory cytokines IL-37 and IL-38, both members of the IL-1 family (Hahn et al., 2017), serve as potent negative regulators of trained immunity. Cavalli et al. demonstrated that IL-37 administration in mice reverses the metabolic and epigenetic reprogramming induced by β-glucan, leading to reduced pro-inflammatory cytokine production (Cavalli et al., 2021). Mechanistically, IL-37 inhibits mammalian target of rapamycin (mTOR) activation, histone modifications such as H3K4me3, and aerobic glycolysis in monocytes and macrophages. These effects translate into diminished immune responses upon secondary stimulation. In parallel, de Graaf et al. showed that recombinant IL-38 prevents the same form of immune training in both murine models and human immune cells, similarly disrupting mTOR signaling and epigenetic activation at inflammatory gene loci, thereby reducing TNF and IL-6 production (De Graaf et al., 2021). Notably, they also identified a human IL1F10 SNP associated with higher IL-38 plasma levels and reduced trained immunity responses. Together, these findings identify IL-37 and IL-38 as endogenous regulators that restrain trained immunity.

IL-1 signaling has been proposed to play a role in trained immunity (Moorlag et al., 2018). Mice trained with β-glucan exhibit higher levels of IL-1β in the blood and enhanced IL-1β production upon exposure to *L. monocytogenes* (Ciarlo et al., 2020). This response was absent in MyD88-deficient mice, indicating that IL-1 signaling through MyD88 (the adaptor molecule downstream of IL-1 receptor) is essential for trained immunity. Furthermore, blockage of IL-1 signaling using an IL-1 receptor antagonist disrupted β-glucan-induced trained immunity in mice (Ciarlo et al., 2020). Collectively, these findings highlight the critical role of IL-1 signaling in mediating the protective effects of trained immunity and demonstrate that its activity can be modulated through receptor blockage.

IL-10 is a well-known anti-inflammatory cytokine, but its role in regulating trained immunity has been less clear. A recent study demonstrated that IL-10 suppresses trained immunity in human monocytes at both functional and transcriptional levels, largely through the STAT3 signaling pathway (Röring et al., 2025). While IL-10 reduced ROS production and dampened glycolytic and oxidative metabolism, it did not reverse β-glucan-induced metabolic shifts or affect phagocytosis. Furthermore, in BCG-vaccinated individuals, IL-10/IL10R genetic variants influence BCG-induced trained immunity, whereas circulating IL-10 levels showed a modest inverse association with changes in ex vivo cytokine production 14 days post-vaccination in men, an effect not observed in women. These findings highlight that IL-10 limits several features of trained immunity, though not all. Elucidating the mechanisms by which it exerts these effects will be crucial for developing IL-10-based strategies to control excessive trained immunity.

The aforementioned mechanisms of negative regulation of trained immunity are likely overlapping and functionally complementary. Further investigation into the broader regulatory network that suppresses trained immunity is needed to deepen our understanding of this biological process.

## Conclusions and perspectives

Trained immunity is a form of long-lasting immunological memory characterized by epigenetic and metabolic reprogramming to a previous stimulus, leading to enhanced responsiveness of innate immune cells to subsequent challenges. TLRs play a key role in initiating trained immunity by recognizing microbial components or endogenous ligands, triggering signaling pathways that induce long-term functional changes in myeloid cells. TLR-mediated trained immunity can enhance the immune response, providing protective immunity against subsequent infections. However, inappropriate activation of this process may contribute to the development of inflammatory and autoimmune disorders. Thus, a better understanding of TLR-mediated trained immunity is pivotal for the design of immunomodulatory drugs that target the mechanisms of trained immunity, which can provide protection against infections and cancer or treat inflammatory diseases.

Strikingly, the majority of studies investigating TLR-trained immunity utilize the TLR4 ligand LPS as a trigger. This preference might be due to the historical recognition of LPS as a potent immuno-stimulator (Alexander and Rietschel, 2001), the fact that it is the only TLR that signals through both the MyD88- and TRIF-dependent signaling pathway and its widespread availability in most laboratories. A more systematic investigation of all TLR ligands, including their dosage and effects on various cell types, as well as a detailed analysis of the downstream pathways and produced immune molecules, is essential to elucidate the full spectrum of mechanisms underlying the establishment of TLR-mediated immunity. In addition, the cellular patterns of TLR expression vary considerably across species, leading to differences in immune signaling and responsiveness. Consequently, the effects of TLR stimulation observed in one species may not translate to another, complicating cross-species comparisons. This variability highlights the necessity for species-specific investigations to accurately assess the impact of TLR priming to trained immunity immune responses. Furthermore, the route of administration of the various TLR agonists has significant effects on the efficiency and magnitude of immune responses, and thus testing and understanding these effects is critical to the success of TLR-mediated trained immunity.

How does the organism decide whether its response should be trained immunity or tolerance to a microbial challenge? We can assume that the decision between trained immunity and tolerance depends on the dose of TLR agonists and the specific cellular context. Low doses of TLR agonists generally promote trained immunity, enhancing the responsiveness of innate immune cells. In contrast, high doses tend to induce tolerance, reducing inflammatory responses. This effect varies from cell type to cell type, due to differences in TLR expression levels and signaling thresholds. Thus, both responses can coexist in an organism, with different cell types potentially developing either trained immunity or tolerance, depending on their specific TLR expression, signaling thresholds, and the local microenvironment.

Despite significant progress, the field of trained immunity still faces knowledge gaps that hinder its clinical translation. Foremost, the precise epigenetic and metabolic signatures that define trained immunity across diverse myeloid and nonmyeloid cell types remain incompletely characterized. Additionally, variability in response among individuals, due to genetic, environmental, and microbiome influences, complicates the predictability and standardization of trained immunity-based interventions. Clinical translation is further challenged by the difficulty in selectively inducing beneficial trained responses without exacerbating inflammation or autoimmunity. Emerging technologies hold the potential to overcome these challenges. Single-cell omics allows unprecedented resolution of cellular heterogeneity and plasticity across training stimuli, while systems biology offers integrative models to predict trained immunity outcomes from multi-omics data. Lineage tracing is advancing our understanding of long-term progenitor reprogramming and the persistence of trained traits across hematopoietic hierarchies. Additionally, nanomedicine can induce trained immunity but also modulate excessive inflammation and correct maladaptive trained immunity across a range of clinical conditions (Van Leent et al., 2022). Together, these technologies promise to refine mechanistic insights into trained immunity and guide precision-targeted strategies to treat infections, chronic inflammatory conditions, autoimmune disorders, cancer, and to enhance vaccine efficiency. Overall, further research defining the precise mechanisms that influence TLR-driven trained immunity and its variability across cell types and species is crucial for optimizing therapeutic approaches that can harness its protective potential while minimizing harmful inflammation.
